# Well-Being and Diabetes Management in Early Pregnant Women with Type 1 Diabetes Mellitus

**DOI:** 10.3390/ijerph13080836

**Published:** 2016-08-22

**Authors:** Karolina Linden, Carina Sparud-Lundin, Annsofie Adolfsson, Marie Berg

**Affiliations:** 1 Institute of Health and Care Sciences, Sahlgrenska Academy, University of Gothenburg, 405 30 Gothenburg, Sweden; carina.s-lundin@fhs.gu.se (C.S.-L.); marie.berg@fhs.gu.se (M.B.); 2 Centre for Person-Centred Care (GPCC), University of Gothenburg, 405 30 Gothenburg, Sweden; 3 School of Health Sciences, Örebro University, 701 82 Örebro, Sweden; annsofie.adolfsson@oru.se; 4 Faculty of Health Sciences, Department of Nursing Science, Vestfold University College of Southeast Norway, 3603 Kongsberg, Norway

**Keywords:** type 1 diabetes mellitus, pregnancy, well-being, diabetes management

## Abstract

This paper explores well-being and diabetes management in women with type 1 diabetes mellitus (DM) in early pregnancy and investigates associations among perceived well-being, diabetes management, and maternal characteristics. Questionnaires were answered by 168 Swedish women. Correlation analyses were conducted with Spearman’s correlation coefficient (r_s_). The women reported relatively high scores of self-efficacy in diabetes management (SWE-DES-10: 3.91 (0.51)) and self-perceived health (excellent (6.5%), very good (42.3%), good (38.7%), fair (11.3%) and poor (1.2%)). Moderate scores were reported for general well-being (WBQ-12: 22.6 (5.7)) and sense of coherence (SOC-13: 68.9 (9.7), moderate/low scores for hypoglycemia fear (SWE-HFS 26.6 (11.8)) and low scores of diabetes-distress (SWE-PAID-20 27.1 (15.9)). A higher capability of self-efficacy in diabetes management showed positive correlations with self-perceived health (r_s_ = −0.41, *p* < 0.0001) and well-being (r_s_ = 0.34, *p* < 0.0001) as well as negative correlations with diabetes distress (r_s_ = −0.51, *p* < 0.0001) and hypoglycemia worries (r_s_ = −0.27, *p* = 0.0009). Women with HbA1c levels of ≤48 mmL/mol scored higher in the subscales “goal achievement” in SWE-DES (*p* = 0.0028) and “comprehensibility” in SOC (*p* = 0.016). Well-being and diabetes management could be supported by strengthening the women’s capability to achieve glycemic goals and their comprehensibility in relation to the treatment. Further studies are needed to test this.

## 1. Introduction

For women with type 1 diabetes mellitus (DM) pregnancy and childbirth is a demanding episode [1]. It is related to increased risk of several complications, such as congenital malformations, miscarriage, large for gestational age, pre-eclampsia, labour inductions, instrumental delivery, neonatal morbidity, stillbirth, and also diabetes-related maternal vascular complications. To give the child the best chance of being born healthy, the primary goal is to achieve near-normoglycemia, which corresponds to a goal level in pregnancy of hemoglobin A1c (HbA1c) ≤ 48 mmol/L [2]. This requires optimal diabetes management including meticulously controlled glycemia, diet, physical activity, and insulin dosages [1,3,4,5].

Well-being in the maternity period for women in general implies a complex interrelationship between simultaneously occurring physiological and psychological changes [6]. As the episodes of pregnancy and childbirth are monumental events recollected for years afterwards [7], the emotional well-being is crucial for women’s health in a lifecycle perspective afterwards. A positive experience facilitates a mother’s personal growth and transformation of self [8]. Many report an increase in self-esteem and a sense of optimism, especially during the second and third trimester of pregnancy [9]. 

In pregnant women with type 1 DM, knowing the increased risk of adverse outcomes, negative feelings of stress, and anxiety over the expectant child have been described. Bodily responses to changes in blood glucose levels alter during pregnancy, and in the struggle to reach near-normoglycemia the women often experience hypoglycemic episodes. Research indicates that the strict insulin regime paired with unfamiliar bodily cues is related to increased vulnerability [1,3,10,11]. Facing the pregnancy-related challenges with this struggle has been identified as being a burden for the pregnant women who fluctuate between mastering and being enslaved by the situation [1,12]. Many women have worked hard to reach recommended near-normoglycemia levels for a long time before even trying to conceive so as to maximize the chances for the child to be born healthy [13,14]. 

A literature review on studies carried out with varied methods in different episodes of pregnancy and after childbirth has concluded that the risk of complications and adverse pregnancy outcomes may have a negative impact on feelings of general well-being in pregnant women with type 1 DM [15]. This literature review is based on studies using either qualitative or statistical methods. As far as we know, statistically designed studies on well-being and diabetes management in early pregnant women with type 1 DM have not yet been published, but should increase the understanding of this complex situation and thereby increase healthcare providers’ caring support to the vulnerable situation for this group of pregnant women. The objective of the study reported in this paper was therefore to (1) explore well-being and diabetes management in women with type 1 DM in early pregnancy and (2) investigate associations among perceived well-being, diabetes management, and maternal characteristics.

## 2. Materials and Methods

The sample of this study was drawn from the Swedish MODIAB-Web (MOtherhood and DIABetes) study: a multi-centre randomized controlled trial (RCT) of women with type 1 DM in pregnancy and early motherhood evaluating the effectiveness, in terms of well-being and diabetes management, of a specially designed web-based support. All the women attended prenatal care at one of the six participating study centres located in the southwestern and central parts of Sweden. More details are described in the published study protocol [16]. The study population in this sub-study consists of a composite group of the control and intervention group. Data was collected after randomization but before the start of the intervention. Ethical approval was obtained from the Ethics Committee of Gothenburg, Sweden (No. 659-09).

In total, 288 women were identified as eligible and invited to participate in the MODIAB-Web study. Of these, 17 did not answer and 95 declined. Of the 176 women who agreed to take part, 2 were never given the baseline questionnaires and 6 did not respond as they had spontaneous or legal abortion, bringing the total number of respondents to 168 women in early pregnancy between November 2011 and December 2014. 

### 2.1. Data Collection

Data consisted of answered questionnaires in early pregnancy (mean 11.2, SD 4.8 weeks of gestation) and of medical and sociodemographic data. The questionnaires consisted of five validated instruments and one single-item Likert scale, three measuring aspects of diabetes management or distress, and three measuring aspects of health and well-being. We consider health and well-being as two interlaced concepts. In addition, medical data was collected from the electronic health record systems and sociodemographics were self-reported by the women.

#### Description of Measurements

The validity and reliability of the instruments in the questionnaire are specified in the study protocol [16]. 

**General well-being** was evaluated through the validated 12-item Well-Being Questionnaire (WBQ12) which is divided into three 4-item subscales; *Negative Well-Being, Energy*, and *Positive Well-Being*. The 12 items can be used to construct an overall scale: General Well-Being (GWB) with a total score of 0–36 where a high score indicates a greater general well-being. No cut-offs have been presented [17,18]. 

**Sense of Coherence** (SOC) was measured through the 13-item validated Sense of Coherence questionnaire (SOC-13) which is divided in three subscales: *Meaningfulness*, *Comprehensibility*, and *Manageability*, giving one score for sense of coherence as a whole and a sub-score for each subscale. Each item is scored on a Likert scale from 1 (low) to 7 (high), giving a possible range of 13–91 [19,20]. Cut-off scores for the total score have been suggested, proposing a score of 60 or less to be considered low, 61–75 moderate and ≥76 to be considered as high [21].

**Self**-**perceived health** was assessed using a single-item Likert scale with the variable values: excellent, very good, good, fair, or poor. The lower the score, the better self-perceived health (EVGFP) [22].

**Self-efficacy in diabetes management** was measured through the Swedish Diabetes Empowerment Scale, the short Swedish version consisting of 10 items (SWE-DES-10). This validated instrument measures psychosocial self-efficacy and is based on the Swedish 23-item version (which originates from the American Diabetes Empowerment scale). Swe-DES-10 consists of 4 subscales (factors): *Goal achievement*, *Self-awareness*, *Stress management* and *Readiness to change*. It uses five point Likert scales ranging from strongly disagree to strongly agree. Higher values indicate stronger empowerment with a total score of 1–5. No general cut-offs have been presented [23,24]. We believe that in order to achieve a high degree of diabetes empowerment a good self-management of diabetes is required, and have therefore chosen to interpret results from this scale as self-efficacy in diabetes management. 

**Diabetes-related distress** was measured through The Swedish-Problem Areas in Diabetes Scale (SWE-PAID-20). This validated instrument produces a total score ranging from 0–100. Patients rate the degree to which each item is currently a problem for them on a Likert scale ranging from “Not a problem” to “Serious problem”. A higher score indicates greater emotional distress. The cut-off score is suggested to be equal to or more than 40 [25,26].

**Hypoglycemia fear** was examined through the validated instrument, The Swedish Hypoglycemia Fear Survey (SWE-HFS), consisting of 20 items divided into three factors: *Hypoglycemia Worry Level*, *Avoidance*, and *Aloneness*. The items are rated on a 5-point Likert scale ranging from “never” to “always” with a total sum score of 0–80. A high score indicates greater hypoglycemia fear but no general cut-offs are presented [27,28].

**Sociodemographic characteristics** were self-reported by the participants. Data included educational level, marital status, occupation, and country of birth. 

Data collected from **medical records** included age, duration of diabetes, the earliest hemoglobin A1c (HbA1c) in mmol/mol (the International Federation of Clinical Chemistry and Laboratory Medicine standard) registered in the pregnancy and if insulin was administered by multiple daily injections or pump. Glycemic control, in terms of HbA1c, can be used as an output measure of diabetes management. For comparative analyses, we have dichotomized HbA1c into two groups, according to the goals as defined in the United Kingdom National Institute for Health and Care Excellence (NICE) guidelines [2]. The NICE guidelines [2] were used as it is close to what is used by clinicians in Sweden, and since Sweden does not have any national guidelines regarding HbA1c goal levels in pregnancy.

### 2.2. Statistical Analysis

Questionnaire responses were all coded by individual participant identification numbers and manually inserted into a data file (SPSS Statistics 22, IBM, Armonk, NY, USA). Continuous variables were described with mean, standard deviation (SD), median, minimum and maximum, and categorical variables with frequencies and percentages. Spearman’s correlation coefficient (r_s_) was used for all correlation analyses. Fisher´s Exact test was used for comparison between groups for dichotomous variables; the Mantel–Haenszel Chi Square Exact test for ordered categorical variables and the Mann–Whitney U-test for continuous variables. Missing data in the 12-item well-being questionnaire (WBQ12) was handled according to the guidelines [29]. In the other instruments, a half-scale approach was undertaken to handle missing data. All tests were two-tailed and conducted at the 5% significance level. Analyses were conducted using SAS System version 9 (SAS Institute, Cary, NC, USA).

## 3. Results

### 3.1. Study Group Characteristics

The mean age of the study population was 30.8 years (range 20–45) with 54.2% expecting their first child. The participants’ mean diabetes duration was 17.0 years (range 0.3–35) and the last measured mean HbA1c of the group was 56 mmol/mol (SD 13) corresponding to 6.4% (SD 1.2), 46 (28%) women had a HbA1c ≤ 48 mml/mol and 119 (72%) had a level above the goal, i.e., ≥49 mmol/mol. Insulin was administered through multiple daily injections in 65.1% of the women; the others used insulin pumps. Nine of the women (5.4%) were born outside of Sweden (mean length of residence in Sweden 14.9 years, range 5–25 years). More characteristics are described in Table 1.

### 3.2. Health, Well-Being, and Diabetes Management

The majority (87.5%) of the women reported excellent, very good, or good self-perceived health (single-item Likert scale: excellent, very good, good, fair, poor (EVGFP)). They gave moderate scores for well-being (WBQ-12) with a mean total score of 22.6 (SD 5.7)), high scores regarding self-efficacy in diabetes management (SWE-DES-10) with a mean total score of 3.91 (SD 0.51)), and low average scores of diabetes distress (SWE-PAID-20) with a mean total score of 27.1 (SD 15.9). More details in relation to the subscales in these questionnaires, as well as for Sense of Coherence (SOC-13) and Hypoglycemia fear (HFS) are reported in Table 2.

### 3.3. Association among Maternal Characteristics, Well-Being, and Self-Efficacy in Diabetes Management

High/positive scores for self-efficacy in diabetes management correlated with a high total score for well-being, sense of coherence, and self-rated overall health and low total scoring for diabetes distress and hypoglycemia fear. The same pattern appeared for associations between general well-being and sense of coherence, self-rated overall health, and diabetes distress (Table 3). A higher degree of well-being was negatively associated with the subscale measuring hypoglycemia worry level (r_s_ −0.21, *p* = 0.012) but not the total score for hypoglycemia fear. 

Small correlations could be found regarding maternal characteristics. Well-being in terms of WBQ-12 was scored higher the older the women were in the total score (r_s_ = 0.17, *p* = 0.031) and lower in the sub-score of negative well-being (r_s_ = 0.25, *p* = 0.0013). The total score of self-efficacy in diabetes management in SWE-DES-10 correlated with older age (r_s_ = 0.19, *p* = 0.015), as did the sub-scores of self-awareness (r_s_ = 0.16, *p* = 0.047) and stress-management (r_s_ = 0.16, *p* = 0.035). The educational level of the participants did not affect the total score of general well-being in WBQ-12 but was significant in the sub-scores of negative well-being where a higher education yielded a lower score (r_s_ = −0.21, *p* = 0.0063) and positive well-being a higher score (r_s_ = 0.18, *p* = 0.023). Parity and diabetes duration was not shown to be associated with well-being (WBQ-12) and self-efficacy in diabetes management (SWE-DES-10) in this study. 

Insulin administration, whether insulin was injected with a pen/syringe or administered with an insulin pump, did not affect the aspects of well-being (WBQ-12) and self-efficacy in diabetes management (SWE-DES-10) except for the sub-score of self-awareness, where participants administering insulin with multiple daily injections scored higher (*p* = 0.036). 

Glycemic control in terms of a lower HbA1c correlated with a higher score in the total score of well-being in terms of WBQ-12 (r_s_ = 0.17, *p* = 0.033) and the sub-score positive well-being (r_s_ = −0.19, *p* = 0.017) and with a lower score of negative well-being (r_s_ = 0.17, *p* = 0.031). Glycemic control correlated with the SWE-DES-10 sub-score goal-achievement (r_s_ = −0.24, *p* = 0.0017) but not with the total score of self-efficacy in diabetes management. This pattern remained when we compared the two groups of HbA1c levels (≤48 vs. ≥49). SWE-DES subscale goal achievement was positively linked with more optimal control (*p* = 0.0028) as was the SOC subscale comprehensibility (*p* = 0.016). No other significant associations were found between HbA1c group and total score/subscales of the questionnaires.

## 4. Discussion 

This study shows that pregnant women with type 1 DM in early pregnancy reported relatively high scores on self-efficacy in diabetes management and self-perceived health, moderate scores of well-being, and low scores of diabetes distress. Higher capability of self-efficacy in diabetes management was positively correlated with self-perceived health and well-being, and negatively correlated with diabetes distress and hypoglycemia worries. 

It is encouraging that the vast majority of the women reported a high degree of health, despite the relatively low sub-score for energy as measured in WBQ-12 (Table 2). This may be explained by the continuous demand to strive for excellent glycemic control [1,3,12] in addition to the usual pregnancy bodily symptoms in early pregnancy such as morning sickness and fatigue. 

The women reported a moderate score for sense of coherence. As we did not have a comparison group in our study design, we do not know if there would have been a difference in the SOC-13 score between our study group and pregnant women without type 1 DM. A Swedish study of 122 healthy pregnant women measuring the SOC 28-item questionnaire in early and late pregnancy, as well as 8 weeks post-partum [30], found that SOC in early pregnancy was a strong predictor for well-being (measured by Health Index and the Hospital Anxiety and Depression scale). The authors suggest that SOC may predict the woman’s capacity to cope with the stress of child-bearing and that a low SOC score may indicate a greater need for professional support during pregnancy. This is supported in our study since higher degree of comprehensibility was found to be linked to better glycemic control.

The reported scores of hypoglycemic fear are in the low third of the scale, suggesting a low to moderate fear of hypoglycemia. The sub-scores regarding worry level and aloneness were similar to those of 384 adult women with type 1 DM in Sweden, although our participants scored lower in terms of fear in situations of being alone [31]. The sub-score of hypoglycemia worry level had a negative correlation with general well-being, but more importantly, the total score correlated negatively with self-efficacy in diabetes management. These findings are noteworthy, as hypoglycemic episodes are said to affect as many as 45% of pregnant women [32]. 

Elements of what could be interpreted as diabetes-related distress (high demands on diabetes management during pregnancy in terms of constant worry, constant pressure, and constant self-blame) have been identified in a qualitative study [1]. It is therefore somewhat encouraging that diabetes-related distress did not seem to be a serious issue for the majority of our study population. Anyhow, health care professionals need to consider the stress of being pregnant and having diabetes. As suggested by Singh et al., one way to balance the message of high risk is to share success stories of previous pregnant women giving birth to healthy children and to point out effective ways of managing risks [33]. It should be kept in mind that the attitude of health professionals may impact the woman’s experience of her pregnancy, and a supportive approach may help facilitate a positive transition to motherhood [15]. The potential distress related to the demands of stricter diabetes management may become compensated by the joy of being pregnant. To our knowledge, diabetes-related distress has not previously been studied quantitatively in a population of pregnant women with type 1 DM. Contemplating that increased anxiety, guilt, and diabetes-related distress were reported in the literature review by Rasmussen et al. [15], it is possible that well-being fluctuates during pregnancy. Nevertheless, our findings are only representative for the first and early second trimester and thus the results are not applicable throughout the pregnancy.

The sub-scale stress management in the Swedish Diabetes Empowerment Scale, the short version (SWE-DES-10), had no significant correlations with any of the used instruments. This instrument has undergone validity and reliability testing in Sweden [23] but it is possible that the sub-scale may not capture stress in pregnant women with diabetes. 

HbA1c in women with optimal levels (≤48 mml/mol) were positively linked with self-efficacy in terms of goal achievement in the SWE-DES subscale (*p* = 0.0028). People with type 1 DM are experts in their own care, and active participation in goal-setting and overcoming barriers leads to better diabetes management and may have a positive impact on blood glucose control [34,35]. A correlation between psychological well-being and glycemia has not been determined [36,37] although poorer perceived health appears to have a negative impact on glycemic control [38]. The median HbA1c in this study population must be considered fairly good at 54 mml/mol with a range of 34–112. A UK-based sample of 1296 early pregnant women (mixed sample of women with both type 1 and type 2 diabetes) in 1996–2008 had a median HbA1c of 62 (range 27–156) [39]. We were unable to compare our findings with any Swedish sample, as HbA1c in early pregnancy is not routinely reported in any national registrar. 

This study showed no significant difference in well-being between women using multiple daily injections and women using insulin pumps, or in terms of parity. To our knowledge, and according to a literature review, no studies have shown differences in terms of health outcomes for mother and child between administrating insulin with a pen or pump [40]. 

It is known that the risks of pregnancy in women with type 1 DM increase with a long duration of diabetes and occurrence of diabetes-related complications [41]. To some extent, a higher educational level and being older both positively correlated with well-being and self-efficacy in diabetes management. This is reminiscent of another study performed in our research group on women with type 1 DM in early motherhood [42]. The reason may be that a higher level of education and greater age leads to personal maturation and to increased preparedness for the new life situation as a mother. Nevertheless, in the present study we found no correlation between diabetes duration and well-being while in the earlier study [42], diabetes duration was an explanatory factor for better well-being at 6 months postpartum. 

### Limitations

Health and well-being are complex phenomena, and so are perceptions of disease management in daily life. The attempt to investigate how these aspects relate to each other is explorative to its character and accomplished by a cross-sectional design and can therefore not say anything about cause and effect. We were not able to find an ideal instrument to measure well-being and diabetes self-management during early pregnancy, and have therefore used six instruments each capturing a different dimension of the phenomena. We acknowledge the great number of correlations made in this paper. The reason for the multiple analyses was to find out how the different aspects related to each other, thereby depicting a pattern of experiences and strategies during a vulnerable and complex life situation.

A central issue to discuss is how representative our study population was. First, it is remarkable that only 58% of the total eligible group did participate. This ratio is not unusual in clinical studies in Sweden, and a possible explanation is that multiple studies are performed simultaneously and therefore compete with each other in terms of recruitment of participants. 

Another limitation is that due to the lack of similar studies measuring well-being and diabetes management in pregnant women with type 1 DM, it is difficult to compare our findings. Compared with a national report of the general maternal population in Sweden 2013, the women’s mean age at childbirth was similar (just above 30 years of age). Regarding education in the general population in the most common maternal age group (30–34), 66% had education beyond secondary school [43] whilst our sample has a similar 65%.

Another limitation of this study is that some of the questionnaires are not commonly used in either general diabetes populations or in general maternity studies, making the results difficult to interpret. Furthermore, it is a methodological weakness that no reference group of healthy pregnant women with a normal progressing pregnancy was used in this study. 

In terms of correlation strength, only small correlations could be found with hypoglycemia fear and maternal characteristics. Most reported correlations were medium or large according to Cohen’s definition [44], although psychosocial variables are typically in the 0.20–0.40 range [45]. 

## 5. Conclusions

This study, investigating associations among perceived well-being, diabetes management, and characteristics in pregnant women with type 1 DM, is hypothesis-generating. From a clinical perspective well-being and self-efficacy in diabetes management could be supported by strengthening the women’s capability and responsibility to achieve the defined goals for HbA1c in pregnancy, and simultaneously inform them that it in early pregnancy it is more common with unfamiliar bodily responses and that the women should, and can only, do as good as they can in striving for near normoglycemia. Furthermore, the clinicians could, together with the woman, implement strategies to overcome the obstacles including approaches to manage fear of hypoglycemic episodes. Since sense of coherence in terms of comprehensibility was significantly associated with better glycemic control, caregivers could support the woman to understand the reason for the different bodily reactions and give support in how to manage daily glycemic ups and downs. All these strategies should be in accordance with a person-centered approach, which emphasizes the individual woman’s narrative, the necessity to make care plans in partnership with the woman, and in order to evaluate this, to document the commonly agreed decisions [46]. What really comprises ideal support to pregnant women with diabetes needs to be furthered investigated.

## Figures and Tables

**Table 1:** Study group characteristics.

Variable	Number	Mean (SD)	Median (Min; Max)
**All participants**	*n* = 168		
**Age**	*n* = 166	30.8 (4.6)	31.0 (20.0; 45.0)
**Educational level**			
Primary school	3 (1.8%)		
Secondary school	55 (33.1%)		
University	108 (65.1%)		
**Parity**			
0	90 (54.2%)		
1	57 (34.3%)		
2	15 (9.0%)		
3	3 (1.8%)		
5	1 (0.6%)		
**Marital status**			
Married/cohabitant	165 (99.4%)		
Single	1 (0.6%)		
**Occupation**			
Employed	136 (81.9%)		
Business owner	3 (1.8%)		
Student	6 (3.6%)		
Unemployed	9 (5.4%)		
Sick leave	7 (4.2%)		
Other	5 (3.0%)		
**Diabetes duration (years)**	*n* = 162	17.0 (8.3)	16.5 (0.3; 35.0)
**HbA1c in early pregnancy (mmol/mol)**	*n* = 165	56 (13)	54 (34; 112)
HbA1c ≤ 48	46 (28%)		
HbA1c ≥ 49	119 (72%)		
**Insulin administration**			
Multiple daily injections	108 (65.1%)		
Pump	58 (34.9%)		

Categorical variables are presented as *n* (%); and continuous variables as Mean (SD)/Median (Min; Max)/*n*.

**Table 2:** Descriptive statistics on instrument scores.

Variable	*N* = 168	Mean (SD)/*n* (%)	Median (Min; Max)
***Well-Being Questionnaire-12 item (WBQ-12)***			
General Well-Being (0–36)	*n* = 168	22.6 (5.7)	23.5 (5.0; 33.0)
Neg Well-Being 12 (0–12)	*n* = 168	3.4 (2.2)	3.0 (0.0; 9.0)
Energy (0–12)	*n* = 168	5.9 (2.7)	6.0 (0.0; 12.0)
Pos Well-Being 12 (0–12)	*n* = 168	8.1 (2.2)	8.0 (0.0; 12.0)
***Sense of Coherence (SOC)-13 item questionnaire***			
SOC: Total Sum (13–91)	*n* = 168	68.9 (9.7)	71.0 (38.0; 88.0)
SOC: Meaningfulness (4–28)	*n* = 168	22.6 (3.1)	23.0 (12.0; 28.0)
SOC: Comprehensibility (5–35)	*n* = 168	25.8 (4.6)	27.0 (11.7; 35.0)
SOC: Manageability (4–28)	*n* = 157	20.6 (3.6)	21.0 (11.0; 27.0)
SOC: Total sum categorized *			
<61		32 (19.0%)	
61–75		88 (52.4%)	
>75		48 (28.6%)	
***Self-perceived Health (EVGFP)***			
Excellent		11 (6.5%)	
Very good		71 (42.3%)	
Good		65 (38.7%)	
Fair		19 (11.3%)	
Poor		2 (1.2%)	
***Swedish Diabetes Empowerment Scale 10-item (SWE-DES-10)***			
SWE-DES-10 Total (1–5)	*n* = 167	3.9 (0.5)	4.0 (2.2; 5.0)
SWE-DES-10 Goal achievement (1–5)	*n* = 167	3.9 (0.7)	4.0 (2.0; 5.0)
SWE-DES-10 Self-awareness (1–5)	*n* = 164	4.1 (0.9)	4.0 (1.0; 5.0)
SWE-DES-10 Stress management (1–5)	*n* = 166	4.0 (0.6)	4.0 (2.0; 5.0)
SWE-DES-10 Readiness to change (1–5)	*n* = 165	3.8 (0.7)	4.0 (2.0; 5.0)
***Swedish-Problem Areas in Diabetes Scale (SWE-PAID-20)***			
SWE-PAID (0–100)	*n* = 148	27.1 (15.9)	23.8 (0.0; 71.3)
SWE-PAID Total sum categorized *			
<40		112 (75.7%)	
≥40		36 (24.3%)	
***Swedish Hypoglycemia Fear Survey (SWE-HFS)***			
HFS Total Sum Score: (0–80)	*n* = 144	26.6 (11.8)	25.0 (4.0; 61.0)
HFS Factor 1: Hypoglycemia worry level (0–40)	*n* = 144	10.3 (7.3)	9.0 (0.0; 28.0)
HFS Factor 2: Avoidance (0–24)	*n* = 168	13.0 (4.0)	13.0 (4.0; 23.0)
HFS Factor 3: Aloneness (0–16)	*n* = 144	3.5 (3.3)	3.0 (0.0; 16.0)

For categorical variables *n* (%) is presented. For continuous variables Mean (SD)/Median (Min; Max)/*n* = is presented. All subjects (*n* = 168). * Categorization according to cut-off levels.

**Table 3:** Correlations between hemoglobin A1c (HbA1c), health, well-being, and self-efficacy in diabetes management or distress.

Label	HbA1c Early Pregnancy	WBQ-12	SWE-DES-10 Total Score
Well-Being Questionnaire-12 item (WBQ-12)	r_s_ −0.17 *		
Sense of Coherence-13 item questionnaire (SOC)	r_s_ −0.23 **	r_s_ 0.59 ***	r_s_ 0.33 ***
Swedish Diabetes Empowerment Scale 10-item (SWE-DES-10)	r_s_ −0.12	r_s_ 0.34 ***	
Self-Perceived Health (Excellent, Very good, Good, Fair, Poor)	r_s_ 0.098	r_s_ −0.54 ***	r_s_ −0.41 ***
Swedish-Problem Areas in Diabetes Scale (SWE-PAID-20)	r_s_ 0.15	r_s_ −0.52 ***	r_s_ −0.51 ***
Swedish Hypoglycemia Fear Survey (SWE-HFS)	r_s_ −0.05	r_s_ −0.11	r_s_ −0.25 **

* *p* < 0.05; ** *p* < 0.01; *** *p* < 0.001 All correlations were calculated with the instruments’ total score.
